# *Salvia connivens* Methanolic Extract Against *Spodoptera frugiperda* and *Tenebrio molitor* and Its Effect on *Poecilia reticulata* and *Danio rerio*

**DOI:** 10.3390/toxics13020094

**Published:** 2025-01-26

**Authors:** Manolo Rodríguez-Cervantes, Luis Ricardo León-Herrera, Salvador Alejandro Ventura-Salcedo, María del Carmen Monroy-Dosta, Eloy Rodríguez-deLeón, Mamadou Moustapha Bah, Juan Campos-Guillén, Aldo Amaro-Reyes, Carlos Eduardo Zavala-Gómez, Rodolfo Figueroa-Brito, Karla Elizabeth Mariscal-Ureta, Héctor Pool, Itzel Ramos-Mayorga, Miguel Angel Ramos-López

**Affiliations:** 1 Facultad de Química, Universidad Autónoma de Querétaro, Querétaro CP 76010, Mexico; 2 Facultad de Ingeniería, Universidad Autónoma de Querétaro, Campus Pinal de Amoles, Querétaro CP 76300, Mexico; 3 Departamento del Hombre y su Ambiente, Universidad Autónoma Metropolitana Unidad Xochimilco, Calzada del Hueso 1100, Coyoacán, Ciudad de México CP 04960, Mexico; 4 Centro de Desarrollo de Productos Bióticos (CEPROBI-IPN), Instituto Politécnico Nacional, Yautepec CP 62731, Mexico; 5 Facultad de Derecho, Universidad Autónoma de Querétaro, Querétaro CP 76010, Mexico; 6 División de Investigación y Posgrado, Facultad de Ingeniería, Universidad Autónoma de Querétaro, Querétaro CP 76010, Mexico

**Keywords:** zebra fish, guppy, fall armyworm, yellow mealworm, rosmarinic acid

## Abstract

*Spodoptera frugiperda* (Lepidoptera: Noctuidae) and *Tenebrio molitor* (Coleoptera: Tenebrionidae) are two prominent pests of maize and its stored grains, respectively. Botanical pesticides have been proposed as an alternative for their management. This study evaluated the insecticidal activity of *Salvia connivens* (Lamiaceae) methanolic extract and rosmarinic acid against *S. frugiperda* and *T. molitor* by adding them to an artificial diet, as well as their ecotoxicological effects on *Poecilia reticulata* (Cyprinodontiformes: Poeciliidae) and *Danio rerio* (Cypriniformes: Danionidae) through acute toxicity tests. The methanolic extract showed higher mortality activity against *S. frugiperda* (LC_50_ = 874.28 ppm) than against *T. molitor* (LC_50_ = 1856.94 ppm) and was non-toxic to fish. Rosmarinic acid, the most abundant compound in the extract (80.45 mg g^−1^), showed higher activity against *S. frugiperda* (LC_50_ = 176.81 ppm). This compound did not cause a toxic effect on adult *P. reticulata* at the tested concentrations. However, in *P. reticulata* fingerlings and *D. rerio* adults, it was non-toxic, except in *D. rerio* embryos, where it was slightly toxic. These findings suggest that *S. connivens* methanolic extract has potential as a botanical product for the management of *S. frugiperda* and *T. molitor* with low ecotoxicological impact, while rosmarinic acid may be a useful compound for the management of *S. frugiperda*.

## 1. Introduction

Pesticides are extensively used in the food and agricultural industries to protect crops during their production, transportation and storage [1]. Unfortunately, due to their extensive and indiscriminate use, they have caused health problems, resistance in the insects they are intended to control and even negative environmental impacts, especially on the atmosphere, soil and water [2,3].

The fall armyworm, *Spodoptera frugiperda*, native to the Americas’ tropical and subtropical regions, has emerged as a significant pest in maize cultivation [4]. The conventional management of this pest involves the use of synthetic insecticides, primarily chlorpyrifos, methomyl and cypermethrin, which belong to the organophosphate, carbamate and pyrethroid chemical groups, respectively [5]. Although these insecticides have been widely used to control *S. frugiperda* populations, they can generate selective resistance in some individuals within a population, allowing them to survive and reproduce, which can lead to the development of insecticide-resistant populations [6,7].

On the other hand, the yellow mealworm, *Tenebrio molitor*, believed to have originated from the Mediterranean region and now found globally, is a pest that infests stored grains [8,9]. Methyl bromide and phosphine are the most employed fumigants for the management of stored-grain pests [10]. The use of methyl bromide is restricted due to its classification as a major ozone-depleting substance. Stored-grain insects such as *Rhyzoperta dominica* (Coleoptera: Bostrichidae) and *Tribolium castaneum* (Coleoptera: Tenebrionidae) have presented resistance to phosphine, the replacement for methyl bromide [11,12]. Additionally, both substances cause neurotoxic and carcinogenic effects in humans [13].

Consequently, there is a need to explore alternative pest management strategies, such as botanical management using aqueous, organic or essential oil extracts from plants [14]. These extracts contain secondary plant metabolites with repellent, antifeedant, developmental inhibitory and insecticidal properties [15]. Furthermore, they are considered safer due to their non-toxicity and environmentally friendly due to their biodegradability, which confers a low to null residuality [16]. According to Damalas et al. (2020) [17], botanical insecticides have proven to be economically viable compared to conventional products for pest management.

*Salvia connivens* is a member of the genus *Salvia*, the largest genus within the Lamiaceae family, comprising roughly 1000 species with a global distribution [18]. *Salvia connivens* is a perennial, herbaceous shrub with blue flowers, native to México. Organic extracts from this plant have revealed the presence of secondary metabolites including alkaloids, flavonoids, terpenoids, lactones, saponins, tannins and carbohydrates [19,20,21]. Chloroform extracts from *S. connivens* have successfully controlled *S. frugiperda* populations, leading to increased mortality and developmental delays, which are manifested as longer larval stages and reduced pupal weight [22]. Other *Salvia* species have demonstrated biological activity against insects. For instance, the essential oil of *Salvia hispanica* (Lamiaceae) has been effective in controlling *Spodoptera exigua* (Lepidoptera: Noctuidae) [23]. Additionally, *Salvia sclarea* (Lamiaceae) essential oil has exhibited insecticidal activity against *Oxycarenus lavatera* (Hemiptera: Oxycarenidae) [24]. Furthermore, the essential oil of *Salvia leriifolia* (Lamiaceae) was found to be toxic to adults of *Lasioderma serricorne* (Coleoptera: Anobiidae) upon contact [25].

Nonetheless, while these extracts have proven effective in controlling pest insects, they may adversely affect non-target species and thus have a detrimental impact on the environment [26]. As highly sensitive organisms, fish exhibit a broad spectrum of responses to environmental changes, including genetic, biochemical, behavioral and morphological alterations, making them excellent bioindicators [27]. Among the most commonly used species for conducting these ecotoxicological studies are *Poecilia reticulata* and *Danio rerio* [26,28].

This study aims to assess the insecticidal activity of the methanolic extract of *S. connivens* and rosmarinic acid against *S. frugiperda* and *T. molitor*, as well as to evaluate their ecotoxicological effects on *P. reticulata* and *D. rerio*.

## 2. Materials and Methods

### 2.1. Preparation of the Extract

Aerial parts (leaves and stems) of wild *S. connivens* plants were collected during the flowering phase in the municipality of Guadalcázar, San Luis Potosí, México (22°39′50.3″ N, 100°24′59.5″). The collection was conducted between 9 and 10 AM (UTC-6) at a site characterized by a clay loam soil texture. The species was authenticated by José García-Pérez at the Isidro Palacios Herbarium of the Autonomous University of San Luis Potosí (Voucher SLPM 43013). Subsequently, the samples were transferred to the Laboratory of Natural Insecticidal Compounds at the Autonomous University of Querétaro (LNIC-AUQ), where they were dried and then pulverized in an IKA WERKE M20 mill (Staufen, Germany). The pulverized plant material was subjected to a reflux extraction at a 1:5 ratio of plant material to solvent. The extract was prepared with J.T. Baker technical-grade methanol (Phillipsburg, NJ, USA) for eight hours and dried with an RV10 rotatory evaporator (Staufen, Germany) [22].

### 2.2. Spodoptera frugiperda Bioassay

The *S. frugiperda* larvae used for this experiment were obtained from the (LNIC-AUQ). Second-instar F4 larvae were used for that bioassay, in line with the methodology proposed by Flores-Macías et al. (2021) [22]. Experimental treatments were administered to the larvae using an artificial diet described by Ramos-López et al. (2010) [29]. To determine the concentrations to be used in the bioassays, a preliminary bioassay was first conducted with five logarithmic concentrations ranging from 0.5 to 5000 ppm. To prepare 150 g of each diet, the dried methanolic extract of *S. connivens* was re-dissolved in distilled water to have a stock solution (0.1 g mL^−1^). Then, aliquots of 7.5 × 10^−4^, 7.5 × 10^−3^, 7.5 × 10^−2^, 7.5 × 10^−1^ and 7.5 mL of the stock solution were incorporated into each diet during its preparation process, along with the diet ingredients and the necessary volume of distilled water considering the volume added with each aliquot. The aim was to establish five concentrations, delimiting between the minimum and maximum biological responses.

The final concentrations used were 500, 1000, 2000, 4000 and 5000 ppm. The amount of 150 g of each diet was prepared following the same process as in the preliminary bioassay. In this case, aliquots of 0.75, 1.5, 3, 6 and 7.5 mL of the stock solution were incorporated into each diet during its preparation. A negative control diet was included, which consisted of an artificial diet without extract.

Each second-instar larva was individually contained in a Primo (Ecatepec, Estado de México, México) no. 0 plastic container, with approximately 1 cm^3^ of artificial diet containing its respective treatment. The larvae were confined in a bioclimatic chamber with a temperature of 27 ± 2 °C, a relative humidity of 70 ± 5% and a photoperiod with a light/dark ratio of 14:10 h. The bioassay was checked daily, with the diet replaced and the excreta cleaned until the pupal stage was reached. During this period, larval mortality and LC_50_ were assessed. A completely randomized experimental design was employed. Each treatment consisted of 20 individuals, divided into 4 replicates of 5 larvae.

### 2.3. Tenebrio molitor Bioassay

The *T. molitor* individuals used in this study were obtained from a colony established at the LNIC-AUQ. Fifth-instar larvae were randomly selected based on the morphological parameters described by Park et al. (2014) [30]. Treatments were administered to the insects through an encapsulated diet developed by our research team [31]. The concentrations to be used were determined through a preliminary bioassay, as previously described for *S. frugiperda*. The concentrations used in this case were 500, 1000, 2000, 4000 and 5000 ppm. For each concentration, 50 mL of encapsulated diet was prepared. The dried methanolic extract of *S. connivens* leaves was re-dissolved with distilled water to create a stock solution (0.05 g mL^−1^). Subsequently, aliquots of 0.5, 1, 2, 4 and 5 mL of this solution were added to each diet during its preparation along with the constituents. All ingredients were mixed until a homogeneous mixture was obtained and subsequently dripped into a 2% calcium chloride solution to produce the encapsulated diet. Finally, the capsules were washed three times with distilled water. A negative control group was included, consisting of a diet without extract.

Each treatment consisted of 20 larvae, which were divided into four replicates of five larvae. Each replicate was individually confined in a polypropylene Petri dish. They were maintained in a bioclimatic chamber with a temperature of 27 °C, with a light/dark photoperiod of 10:14 h and a relative humidity of 40%, based on the conditions proposed by Mirzaeva et al. (2020) [32]. Once the bioassay was established, it was checked daily, with the diet replaced and exuviae and dead insects removed. Observations were conducted until day 7 when 100% mortality was detected in the larvae exposed to the maximum concentration.

### 2.4. Fish Acclimation and Conditioning Process

The specimens of *P. reticulata* and *D. rerio* were provided from a stable colony established at the Live Feed Chemical Analysis Laboratory of the Autonomous Metropolitan University, Xochimilco Unit. The fish were acclimated for 14 d, being re-placed in 90 cm long × 40 cm wide × 30 cm high glass aquariums, with aquatic plants (*Elodea* sp.) introduced to avoid fish stress. In a semi-hard reconstituted water medium (pH: 7.4–7.8; hardness: 80–100 mg L^−1^ as CaCO_3_) [33], the fish were fed with Grupo Acuario Lomas (Ciudad de México, México) fish flakes. The temperature of the aquarium water was 28 ± 3 °C.

### 2.5. Fish Ecotoxicity Bioassay

The toxicological evaluation was conducted on male *P. reticulata* adults and fingerlings, as well as on embryos and male adults of *D. rerio*, as described by Martínez and Espinoza (2008) [34]. The evaluated concentrations of the methanolic extract of *S. connivens* were 500, 250, 125, 62.5, 31.2 and 0 mg L^−1^. To prepare 200 mL of each solution, the dried extract was re-dissolved in semi-hard water for fish rearing to produce a stock solution (3.875 mg mL^−1^). Subsequently, aliquots of 25.8, 12.9, 6.45, 3.22 and 1.61 mL of this solution were individually brought up to a final volume of 200 mL with semi-hard water. The negative control consisted solely of this water.

For the *P. reticulata* experiments, adults aged 75–80 d post-partum were used, while fingerlings were 5–6 d old. On the other hand, for the *D. rerio* experiments, adults aged between 85 and 90 d since hatching were employed. Twelve individuals per concentration were divided into four replicates of three fish. Each replicate was placed in a 250 mL circular plastic container with 200 mL of the corresponding extract solution. The container size was selected because it is suitable for short-term tests. Definitive readings of toxicity tests were taken at 1, 3, 6, 12, 24, 48, 72 and 96 h of exposure, with mortality values recorded when no opercular movement was observed. The fish were not fed during the bioassays. The 96 h mortality data were used to estimate the LC_50_ values.

The *D. rerio* embryos were exposed individually in 24-well microplates. Embryos were treated 24 h after fertilization for a duration of 96 h [35]. For each treatment, 16 embryos were used and divided into four replicates of four embryos. Each embryo was individually placed in a well containing 2 mL of the corresponding extract.

The Bioethics Committee of the Faculty of Chemistry at the Autonomous University of Querétaro (FC-AUQ) approved all bioassays conducted in this study under Register Number CBQ24/094.

Fish that survived the experiment were euthanized by immersion in ice water [36]. Subsequently, biological waste (dead fish) was managed in accordance with the NOM-087-SEMARNAT-SSA1-2002 [37] standard and the hazardous waste agreement held by the FC-AUQ with a private company. The fish were properly stored in yellow polyethylene bags marked with the biohazard symbol for biological waste and then placed in a freezer designated for this purpose and made available to the company contracted by the faculty.

The water contaminated with the extracts previously described in the methodology was properly stored in plastic drums classified as organic waste. Subsequently, these drums were transferred to the Faculty of Chemistry’s temporary hazardous waste storage facility for subsequent management by a specialized private company.

### 2.6. Identification of the Major Compound in the Extract

Analyses were performed on a Waters Alliance HPLC system (Milford, CT, USA) equipped with a model e2695 pump and a model 2998 diode array detector (DAD). Data acquisition and processing were carried out using Empower 3 (Chromatography Data System, CDS).

For the identification and quantification of the rosmarinic acid in the extract, the chromatographic conditions consisted of a C18 column (5 µm, 150 × 4.5 mm) and a mobile phase composed of water acidified with 12.5 mM acetic acid and acetonitrile. An elution gradient was employed for the analysis of rosmarinic acid, utilizing a mobile phase comprising 12.5 mM acetic acid and acetonitrile. The gradient started with 95% acetic acid and 5% acetonitrile for the initial 2 min. A linear increase in acetonitrile content to 50% over 15 min (from 5 to 20 min) was then implemented. Subsequently, the mobile phase was rapidly reverted to the initial conditions with 95% acetic acid and 5% acetonitrile at 25 min, which were maintained for the final 5 minutes of the 30 min analysis. A flow rate of 1 mL min^−1^ was used. Detection was performed at a wavelength of 328 nm, with 10 µL of sample injected. A calibration curve was established using a series of rosmarinic acid standard solutions (Merck, 96%, Naucalpan de Juárez, Mexico) with concentrations of 25, 50, 100, 125 and 200 µg mL^−1^.

### 2.7. Assessment of the Insecticidal Activity and Ecotoxicological Impact of the Major Compound

The insecticidal activities of rosmarinic acid against *S. frugiperda* and *T. molitor* were assessed using concentrations of 1000, 600, 400, 160, 80 and 0 ppm. The bioassay protocol previously established for the methanolic extract of *S. connivens* was followed, adding the concentrations of rosmarinic acid for each diet during their preparation. For the *S. frugiperda* diets, aliquots of 1.5, 0.9, 0.6, 0.24 and 0.12 mL of a stock solution of rosmarinic acid (0.1 g mL^−1^) were added to each diet during its preparation, in order to produce 150 g of each concentration. In the case of *T. molitor* diets, 50 mL of each diet was made adding 1, 0.6, 0.4, 0.16 and 0.08 mL of a stock solution of rosmarinic acid (0.05 g mL^−1^) during their production process.

For the evaluation of ecotoxicological effects, *P. reticulata* (male adults and fingerlings) and *D. rerio* adults, as well as *D. rerio* embryos, were exposed to 500, 250, 125, 62.5, 31.2 and 0 ppm of rosmarinic acid, following the same methodology previously described. A stock solution of rosmarinic acid (3.875 mg mL^−1^) was prepared with semi-hard water. Subsequently, aliquots of 25.8, 12.9, 6.45, 3.22 and 1.61 mL of this solution were individually brought up to a final volume of 200 mL with semi-hard water. A negative control group was included, consisting solely of water.

### 2.8. Statistical Analysis

The data collected from the assessment of the insecticidal and ecotoxicological activity were analyzed using one-way analysis of variance (ANOVA) followed by Tukey’s test to identify significant differences among means. A probit analysis was conducted to calculate the LC_50_. All analyses were performed using SYSTAT 9 statistical software [38] at a 95% confidence level.

## 3. Results

### 3.1. Insecticidal Activity of the Methanolic Extract of S. connivens Against S. frugiperda and T. molitor

The larval mortality was found to be concentration-dependent in both biological models (Table 1). In the case of *S. frugiperda*, a significant response compared with the control was observed starting at 500 ppm, with 60% mortality. This was followed by responses of 65, 80 and 85% at concentrations of 1000, 2000 and 4000 ppm, respectively. Meanwhile, in *T. molitor*, significant larvicidal activity was observed starting at 1000 ppm, causing 50% larval mortality. At a concentration of 2000 ppm, the mortality remained at 50%. However, this increased to 85% in the 4000 ppm treatment. When both insects were treated with 5000 ppm of the extract, 100% larval mortality was recorded.

The LC_50_ value for *S. frugiperda* was found to be lower than that for *T. molitor*, with those values being 874. 28 and 1856.94 ppm, respectively.

### 3.2. Ecotoxicological Effect of the Methanolic Extract of S. connivens on P. reticulata and Danio rerio

The methanolic extract of the *S. connivens* aerial parts exhibited toxic effects on the adult stages of both *P. reticulata* and *D. rerio*, as well as the *D. rerio* embryos. Adult *P. reticulata* exposed to the extract for 96 h showed cumulative mortalities of 16.67, 25, 33.33, 83.33 and 100% at concentrations of 31.2, 62.5, 125, 250 and 500 ppm, respectively (Table 2).

Mortality was first observed after 3 h of exposure. The calculated LC_50_ value for the adult *P. reticulata* was 153.10 ppm (Table 2).

The adult *D. rerio* demonstrated similar toxicity patterns, with cumulative mortalities of 8.33, 16.66, 100 and 100% at concentrations of 62.5, 125, 250 and 500 ppm, respectively (Table 3). Mortality was observed as early as the first hour of exposure. The LC_50_ value for the adult *D. rerio* was 154.32 ppm.

In the *D. rerio* embryos, the extract exhibited cumulative mortalities of 25, 25, 25, 50 and 100% at concentrations of 31.2, 62.5, 125, 250 and 500 ppm, respectively. Mortality onset occurred after 3 h of exposure. The calculated LC_50_ value for the embryos was 208.38 ppm.

### 3.3. Identification of Rosmarinic Acid

**Figure 1:**
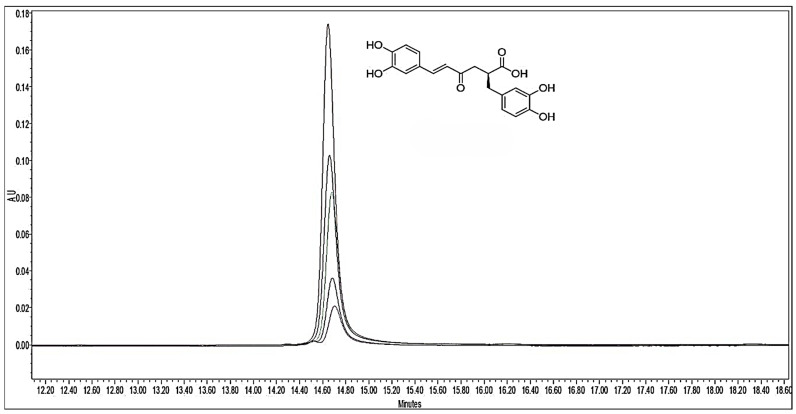
Chromatogram of the rosmarinic acid standard at different concentrations for the calibration curve.

The rosmarinic acid standard exhibited a retention time in a range of 14.649 to 14.698 min and a maximum absorbance at a wavelength of 328 nm. Figure 1 presents the standard response obtained in the range of 25 to 200 µg mL^−1^, used to construct the calibration curve for the quantification of this compound. The high coefficient of determination (R^2^ = 0.998) obtained for this calibration curve confirms a strong linear relationship between the peak area and the concentration of rosmarinic acid.

**Figure 2:**
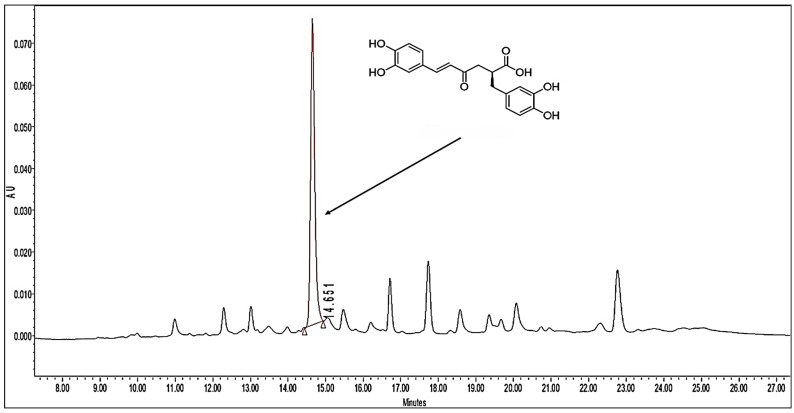
Chromatogram of the *Salvia connivens* methanolic extract. The arrow indicates the signal of rosmarinic acid.

The chromatogram of the *S. connivens* methanolic extract (Figure 2) revealed a peak corresponding to rosmarinic acid with a retention time of 14.651 min. This peak displayed the highest intensity among all detected compounds, suggesting that the rosmarinic acid was a predominant component of the extract. The quantitative analysis indicated a rosmarinic acid concentration of 80.45 mg g^−1^ of extract.

### 3.4. Insecticidal Activity of Rosmarinic Acid Against S. frugiperda and T. molitor

In the case of *S. frugiperda*, the rosmarinic acid induced significant mortality compared with the control, starting at 80 ppm, with a mortality rate of 45% (Table 4). Subsequently, mortalities of 55, 85 and 95% were observed at 160, 400 and 600 ppm, respectively. Furthermore, the total mortality of the population was observed at 1000 ppm. The LC_50_ value for *S. frugiperda* was 176.81 ppm. In contrast, the rosmarinic acid induced only 10% mortality at 1000 ppm in *T. molitor*. An LC_50_ of 5256.28 ppm was estimated for this species, indicating a lower insecticidal activity compared to the LC_50_ value that *S. frugiperda* (176.81 ppm) showed in this research.

### 3.5. Ecotoxicological Effect of Rosmarinic Acid on P. reticulata and D. rerio

No mortality effects were observed in adult *P. reticulata* exposed to rosmarinic acid at any of the tested concentrations or time points (Table 5). Consequently, no LC_50_ value could be calculated.

The *Poecilia reticulata* fingerlings exhibited mortality rates of 8.33, 16.66 and 25% at concentrations of 125, 250 and 500 ppm, respectively. The first signs of mortality were observed after 24 h of exposure. The calculated LC_50_ value for the fingerlings was 658.88 ppm.

The adult *D. rerio* also showed accumulated mortalities of 8.33, 8.33 and 58.33% at concentrations of 125, 250 and 500 ppm, respectively (Table 6). Mortality was first observed after 48 h of exposure. The LC_50_ value for the adult *D. rerio* was 463.81 ppm.

In contrast, the *D.* rerio embryos demonstrated a significantly higher sensitivity to rosmarinic acid. At concentrations of 31.2 and 62.5 ppm, the mortality reached 93.75%. Higher concentrations (125, 250 and 500 ppm) resulted in 100% mortality. In this case, the first record of mortality was observed at 3 h. The calculated LC_50_ value for the embryos was 21.42 ppm.

## 4. Discussion

### 4.1. Insecticidal Activity of the Methanolic Extract of Salvia connivens Against Spodoptera frugiperda

The insecticidal activity of plants belonging to the genus *Salvia* has already been reported. Pavela and Chermenskaya (2004) [39] evaluated the biological activity of methanolic extracts from the aerial parts of different plants against *Spodoptera littoralis* (Lepidoptera: Noctuidae). The evaluated species included *Salvia officinalis* and *Salvia splendens* (Lamiaceae), which showed LC_50_ values of 4.7 and 77.1 ppm, respectively. Nonetheless, Kamaraj et al. (2008) [40] reported insecticidal properties from other Lamiaceae species, such as *Ocimum canum* and *Ocimum sanctum* (Lamiaceae). In their study, they found that *Spodoptera litura* (Lepidoptera: Noctuidae) larvae fed on leaves treated with the methanolic leaf extracts of *O. canum* and *O. sanctum* at 1000 ppm experienced mortality rates of 100 and 71%, respectively, with the *O. canum* showing an LC_50_ value of 36.46 ppm. Additionally, Sakr and Roshdy (2015) [41] reported that the methanolic extract of *Hyptis brevipes* (Lamiaceae) caused 85 and 100% mortalities against *S. littoralis* larvae at concentrations of 12,500 and 50,000 ppm, respectively. Furthermore, their study revealed significant histological alterations in vital organs such as the midgut and malpighian tubules, which they attributed to the secondary metabolites present in *H. brevipes*.

The results obtained in the present study differ from those reported in previous research. While those studies found both higher and lower LC_50_ values, all have agreed on the insecticidal activity of plants from the genus *Salvia* and the Lamiaceae family against noctuid insect pests.

The variations in the mortality rates among the studies may be due to differences in the secondary metabolite profiles of the plants, which can be influenced by species, environment and genetics [42]. The insecticidal activity of plant extracts can also vary considerably, even within the same genus. According to Zavala-Sánchez et al. (2013) [43], the chloroform extracts of *Salvia microphylla* (Lamiaceae) and *S. connivens* (LC_50_ = 916 and 936 ppm, respectively) showed higher insecticidal activity against *S. frugiperda* compared to *Salvia keerlii* and *Salvia ballotiflora* (Lamiaceae) (LC_50_ = 1527 and 1685 ppm, respectively). The results presented in this study show similarities to those reported by Zavala-Sánchez et al. (2013), although some variability was also observed. This variability could be attributed to the different polarities of the solvents used, which led to the extraction of different types of compounds. In another study, the methanolic extract of *Ajuga iva* (Lamiaceae) was evaluated against *S. littoralis* larvae, with up to 87% mortality observed at a concentration of 250,000 ppm [44]. Furthermore, the aqueous extract of *S. microphylla* exhibited insecticidal activity against *Aphis pomi* (Hemiptera: Aphididae), inducing up to 73.33% mortality at a concentration as high as 100,000 ppm, with an LC_50_ value of 70,090 ppm [45].

These data support the idea that *Salvia* species can be effective against a variety of pest insects, including those outside the Noctuidae family. However, the organic extracts of the *Salvia* species have generally demonstrated superior insecticidal efficacy compared to the aqueous extracts.

### 4.2. Insecticidal Activity of the Methanolic Extract of Salvia connivens Against Tenebrio molitor

There is limited research on the insecticidal activity of *Salvia* species against *Tenebrio molitor*, although some studies have investigated the insecticidal properties of other Lamiaceae species against a variety of stored-grain insects. Marouf et al. (2008) [46] demonstrated that the methanolic extract of *Origanum vulgare* (Lamiaceae) induced 30.38% mortality in *Tribolium confusum* (Coleoptera: Tenebrionidae) larvae at 2138 ppm (LC_50_ = 2578.07 ppm) and 45.93% mortality in *Callosobruchus maculatus* (Coleoptera: Chrysomelidae) at 4365 ppm (LC_50_ = 4416.13 ppm) after 3 d. The activity of the methanolic extract of *O. canum* leaves against *C. maculatus* adults has also been studied; a concentration of 5000 ppm caused 66.20% mortality after 7 d [47]. Meanwhile, Jbilou et al. (2008) [48] also tested the insecticidal activity of methanolic extracts from seven plant species against *T. castaneum* larvae, including *A. iva*, which experienced significant larval mortality of 31% at a concentration of 100,000 ppm. Aqueous extracts have also been tested against pests that attack cereal grains, such as the extract of *Salvia rosmarinus* (Lamiaceae) against *Zabrus tenebrioides* (Coleoptera: Carabidae) larvae, which presented an LC_50_ value of 8323.02 ppm after 8 d of exposure [49].

The results of the present study are comparable with those obtained by Marouf et al. (2008), Kosini et al. (2015) and Khidr et al. (2024), with some notable differences. While the mortality rates observed in this study were higher than those in most previous studies, Jbilou et al. (2008) observed mortality at significantly higher concentrations (100,000 ppm) than those in our findings.

This indicates that polar extracts, such as methanolic ones, from Lamiaceae species, particularly those belonging to the genus *Salvia*, could also provide effective and sustainable tools for managing stored-grain pests.

### 4.3. Ecotoxicological Effect of the Methanolic Extract of Salvia connivens on Poecilia reticulata and Danio rerio

Few ecotoxicological studies have assessed the effects of botanical products from Lamiaceae species on fish. de Oliveira et al. (2022) [50] reported an LC_50_ value of 924.89 ppm for *Tetradenia riparia* (Lamiaceae) essential oil against adults of *Gambusia affinis* (Cyprinodontiformes: Poeciliidae). Additionally, Govindarajan et al. (2016) [51] assessed the ecotoxicity of *Origanum scabrum* (Lamiaceae) essential oil in *G. affinis*, reporting an LC_50_ value of 12425.66 ppm.

Similarly, the toxicities of organic extracts have been studied in non-target fish. Silvagnaname and Kalyanasundarum (2004) [52] found LC_50_ values of 23.36 and 20.62 ppm for *G. affinis* and *P. reticulata*, respectively, using a methanolic extract of *Atlantia monophylla* (Rutaceae) leaves. Patil et al. (2011) assessed the toxicities of ethanolic and dichloromethane extracts from *Plumbago zeylanica* (Plumbaginaceae) and *Cestrum nocturnum* (Solanaceae), respectively. *Plumbago zeylanica* was the only species that caused mortality in *P. reticulata*, with values of 10 and 20% at 27.4 and 72.06 ppm, respectively. In contrast, Ravindran et al. (2020) [53] evaluated the ecotoxicity of the methanolic extract of *Clitoria ternatea* (Fabaceae) flowers and found no mortality in *P. reticulata*, even at concentrations as high as 2500 ppm. Hernández-Caracheo et al. (2023) [26] assessed the ecotoxicities of acetone and methanol extracts from the aerial parts of *Heterotheca inuloides* (Asteraceae) in *P. reticulata*. They reported LC_50_ values of 12.39 and 62.49 ppm for fingerlings and adults, respectively, when exposed to the acetone extract. The methanol extract was less toxic, with LC_50_ values of 186.16 and 223.97 ppm.

The results obtained in this study exhibit considerable variability when compared with those reported by other authors. This discrepancy could be attributed to differences in the plant species employed, the animal models used and the types of compounds evaluated (essential oils and extracts). However, the findings of Silvagnaname and Kalyanasundarum (2004) suggest that the observed variations are linked not necessarily to the biological model used but rather to the chemical compositions of the botanical extracts. In fact, those authors reported a higher toxicity for the *A. monophyla* extract compared with the results of the present study. On the other hand, the results of Patil et al. (2011) and Hernández-Caracheo et al. (2023) show greater concordance with ours. Additionally, according to Hernández-Caracheo et al. (2023), methanolic extracts are generally less toxic to fish than acetonic ones.

It is important to highlight that, according to the pesticide toxicity classification for aquatic animals described by Helfrich et al. (2009) [54], most of the extracts evaluated in this study and in the literature consulted are classified as “non-toxic” to aquatic organisms.

While toxicity studies of plant extracts within the order Lamiales are relatively common, particularly in the Verbenaceae family, there is a notable dearth of similar research on Lamiaceae species with *D. rerio*. *Lippia sidoides* (Verbenaceae); previous studies have shown no toxicity in adult *D. rerio* after 96 h of exposure to a concentration of up to 400 ppm of its aqueous extract, as reported by Camilo et al. (2022) [55]. Similarly, Nonato et al. (2023) [56] evaluated the aqueous and ethanolic extracts of *Lippia alba*, *Lippia sidoides* and *Lippia gracilis* (Verbenaceae). They found no mortality at concentrations of up to 400 ppm after 96 h of exposure, indicating a lack of toxicity in these species as well. However, the ecotoxicities of plant extracts from families other than Lamiales have also been assessed in *D. rerio*, as seen in a study by Xavier and Kripasana (2020) [57], who assessed the effect of the ethanolic extract of *Enydra fluctuans* (Asteraceae) leaves on that organism and observed an LC_50_ value of 92.65 ppm.

Studies have also been conducted on the effects of extracts from Lamiaceae species on *D. rerio* embryos. For example, Abidin et al. (2023) [58] obtained an LC_50_ value of 7.68 ppm with the ethanolic extract of *Vitex trifolia* (Lamiaceae) leaves. On the other hand, Nguyen et al. (2021) [59] evaluated the embryotoxic effects of the ethanolic leaf extract of *Clerodendron crytophyllum* (Lamiaceae), reporting an LC_50_ value of 79.61 ppm. In contrast, Ullah et al. (2024) [60] found a lower LC_50_ value, of 5 ppm, when testing the methanolic extract of *Marrubium vulgare* (Lamiaceae).

According to these studies, plant extracts from the Verbenaceae family, belonging to the Lamiales order, exhibit non-toxicity in adult *D. rerio* and are considered non-toxic based on the pesticide toxicity classification: a finding consistent with our current study. In contrast, Xavier and Kripasana (2020) reported considerably different results, categorizing the extract of *E. fluctuans* as slightly toxic. Moreover, studies evaluating Lamiaceae species in embryos have shown greater variability. These species are generally categorized as moderately and slightly toxic, while the extract in the present study was found to be non-toxic.

Therefore, the botanical extracts used for pest management are generally safe for the environment and human health; because of this, they are considered eco-friendly. In particular, the methanolic extract of *S. connivens* was found to be non-toxic using acute toxicity tests on *P. reticulata* and *D. rerio*.

### 4.4. Identification and Quantification of Rosmarinic Acid

Previous research has identified the presence of rosmarinic acid in *Salvia* species. Zenghin et al. (2018) [61] used High-Performance Liquid Chromatography with Electrospray Ionization Mass Spectrometry Detection (HPLC-ESI-MS) to characterize the phenolic profiles of *Salvia blepharochlaena*, *Salvia euphratica* and *Salvia verticillate* (Lamiaceae) methanolic extracts. Rosmarinic acid was found to be the most abundant compound in all three species, with concentrations of 22, 10.3 and 67 mg g^−1^, respectively. Furthermore, Al-Jaber et al. (2020) [62] profiled the phenolic and flavonoid compounds in the methanolic extracts of *Salvia eigii*, *Salvia hierosolymitana* and *Salvia viridis* (Lamiaceae) through HPLC-ESI-MS, obtaining concentrations of 15.78, 27.12 and 0.32 mg g^−1^ of extract, respectively. They found that rosmarinic acid was the most abundant compound in *S. eigii* and *S. hierosolymitana*. In contrast, Jahani et al. (2022) [63] employed HPLC-UV to quantify rosmarinic acid in the methanolic extract of *Salvia limbate* (Lamiaceae), reporting a concentration of 120.28 mg g^−1^. Similarly, Paje et al. (2022) [64] profiled the phenolic acids and flavonoids of four *Salvia* species using HPLC-UV and found rosmarinic acid contents of 1.58, 1.67, 2.13 and 48.22 mg g^−1^ in the methanolic extracts of *S. officinalis, Salvia splendens*, *Salvia japonica* and *Salvia plebeian* (Lamiaceae), respectively. Rosmarinic acid was the major compound in *S. officinalis*, *S. splendens* and *S. japonica*.

Consistent with these previous findings, rosmarinic acid was the predominant compound in the methanolic extract of the *S. connivens* aerial parts. Nevertheless, rosmarinic acid concentrations vary significantly among *Salvia* species. The levels detected in our study align more closely with those reported for *S. verticillate* and *S. limbate* by Zenghin et al. (2018) and Jahani et al. (2022), respectively.

### 4.5. Insecticidal Activity of Rosmarinic Acid Against Spodoptera frugiperda and Tenebrio molitor

The insecticidal potential of rosmarinic acid has been explored in some studies. Mughees et al. (2021) [65] reported LC_50_ values of 42.41 and 23.55 ppm for *R. dominica* and *Batrocera dorsalis* (Diptera: Tephritidae), respectively, while showing no toxicity to *Planococcus citri* (Hemiptera: Pseudococcidae). Khan et al. (2019) [66] found a much lower LC_50_ value, of 0.2 ppm, for *Acrythosiphon pisum* (Hemiptera: Aphididae).

Additional studies have explored the insecticidal properties of other phenolic compounds. Punia et al. (2021) [67] reported an LC_50_ value of 402.8 ppm for gallic acid against *S. litura*. Guerra et al. (1990) [68], on the other hand, found mortalities of 28.6, 31.5 and 33.5% against *Heliothis zea* (Lepidoptera: Noctuidae) at concentrations of 200 ppm of trans-cinnamic acid, catechin and catechol, respectively.

Previous studies have highlighted the potential of rosmarinic acid and other phenolic compounds for pest control, although their results vary from those presented in this study. Those presented by Mughees et al. (2021) [65] and Khan et al. (2019) [66] show that species from different orders exhibit variability in susceptibility to this compound, with higher susceptibility or even no mortality in some insects, such as *T. molitor* in the present work. In studies conducted with noctuids, it has been shown that they require higher concentrations of this class of compounds to elicit a biological response than those required against Spodoptera frugiperda in this work.

The results obtained demonstrate a marked difference in susceptibility to rosmarinic acid between *S. frugiperda* and *T. molitor*. This finding highlights the importance of considering species-specific factors when evaluating the insecticidal activity of natural compounds. Further research is warranted to investigate the underlying mechanisms of this differential sensitivity and to explore potential strategies for optimizing the insecticidal activity of rosmarinic acid against specific target pests.

### 4.6. Ecotoxicological Effect of Rosmarinic Acid on Poecilia reticulata and Danio rerio

Rosmarinic acid elicited different responses in both organisms in this study. In adult *P. reticulata*, it did not induce toxicity at the evaluated concentrations, whereas it did in juveniles as well as in adult *D. rerio*, although it would still be considered non-toxic. In embryos, it would be considered slightly toxic.

Previous studies have evaluated the toxicities of phenolic compounds in aquatic organisms. Caffeic acid has demonstrated a high LC_50_ value (>100 ppm) in *Cyprinus carpio* (Cypriniformes: Cyprinidae), as reported by Craioveanu et al. (2014) [69]. In addition, Techer et al. (2015) [70] found an LC_50_ value of 707 ppm for adult *D. rerio* exposed to gallic acid.

Regarding embryotoxic effects, Harishkumar et al. (2019) [71] assessed the toxicities of quercetin, gallic acid and curcumin, finding LC_50_ values of 484, 303 and 135 ppm, respectively, in *D. rerio* embryos. Resveratrol, on the other hand, has shown an LC_50_ value of 75.3 ppm in *D. rerio* embryos, as reported by Cavalcante et al. (2017) [72].

Our findings diverge from those obtained with other phenolic compounds, yet all compounds were classified as non-toxic. Our embryo results, however, align more closely with those of Cavalcante et al. (2017), falling within the “slightly toxic” category. These data suggest that rosmarinic acid could be a promising candidate for pest management, particularly against *S. frugiperda*, while posing minimal environmental risk.

## 5. Conclusions

The methanolic extract of the aerial parts of *S. connivens* exhibited insecticidal activity against *S. frugiperda* and *T. molitor*, with greater efficacy against *S. frugiperda*. Ecotoxicity assays revealed that the extract was non-toxic to adults and juveniles of *P. reticulata*, as well as adult and embryonic *D. rerio*, based on the calculated LC_50_ values.

The identification of rosmarinic acid as the predominant compound in the extract and its insecticidal activity against *S. frugiperda* were confirmed. The acid did not show significant insecticidal activity against *T. molitor*. This compound was non-toxic to the adult *P. reticulata* and adult *D. rerio*, although it exhibited slight toxicity toward the *D. rerio* embryos.

Therefore, the methanolic extract of *S. connivens* can be considered a botanical product useful for the management of *S. frugiperda* and *T. molitor* with low ecotoxicological impact, while rosmarinic acid may be a useful compound for the management of *S. frugiperda* alone.

## Figures and Tables

**Table 1:** Insecticidal activity of the methanolic extract of *Salvia connivens* against *Spodoptera frugiperda* and *Tenebrio molitor*.

Treatment (ppm)	Larval Mortality (%)	
	*S. frugiperda*	*T. molitor*
5000	100 ± 0 ^A^	100 ± 0 ^A^
4000	85 ± 8.19 ^AB^	85 ± 8.19 ^A^
2000	80 ± 9.18 ^AB^	50 ± 11.5 ^B^
1000	65 ± 10.9 ^B^	50 ± 11.5 ^B^
500	60 ± 11.2 ^B^	25 ± 9.18 ^BC^
0	5 ± 5 ^C^	0 ± ND ^C^
LC_50_	874.28 (330.02–1418.55) ppm	1856.94 (1417.83–2296.06) ppm

Results are the average of 20 measurements ± standard error. Different letters indicate significant differences. LC_50_ is shown with its confidence intervals. ND stands for not determinable.

**Table 2:** Ecotoxicological effect of the methanolic extract of *Salvia connivens* on *Poecilia reticulata* adults and fingerlings.

***P. reticulata* Adults**									
**Treatment (ppm)**	**Time (h)**								
	**1 h**	**3 h**	**6 h**	**12 h**	**24 h**	**48 h**	**72 h**	**96 h**	**Total**
500	0	100	-	-	-	-	-	-	100 ± 0 ^A^
250	0	25	0	41.65	8.33	0	8.33	0	83.33 ± 11.2 ^A^
125	0	0	0	0	0	16.66	16.66	0	33.33 ± 14.2 ^B^
62.5	0	0	0	0	8.33	8.33	8.33	0	25 ± 13.1 ^B^
31.2	0	0	0	8.33	0	8.33	0	0	16.66 ± 11.2 ^B^
0	0	0	0	0	0	0	0	0	0 ± ND ^B^
LC_50_	153.10 (109.41–196.78) ppm								
***P. reticulata* Fingerlings**									
**Treatment (ppm)**	**Time (h)**								
	**1 h**	**3 h**	**6 h**	**12 h**	**24 h**	**48 h**	**72 h**	**96 h**	**Total**
500	0	75	25	-	-	-	-	-	100 ^A^
250	0	0	0	0	50	25	0	0	75 ^B^
125	0	0	0	0	0	0	0	0	0 ^C^
62.5	0	0	0	0	0	0	0	0	0 ^C^
31.2	0	0	0	0	0	0	0	0	0 ^C^
0	0	0	0	0	0	0	0	0	0 ^C^
LC_50_	238.01 (ND) ppm								

Results represent the average of 12 measurements. Total results are shown ± standard error. Different letters indicate significant differences. LC_50_ is shown with its confidence intervals. ND stands for not determinable.

**Table 3:** Ecotoxicological effect of the methanolic extract of *Salvia connivens* on *Danio rerio* adults and embryos.

***D. rerio* Adults**									
**Treatment (ppm)**	**Time (h)**								
	**1 h**	**3 h**	**6 h**	**12 h**	**24 h**	**48 h**	**72 h**	**96 h**	**Total**
500	75	25	-	-	-	-	-	-	100 ± 0 ^A^
250	0	0	50	50	-	-	-	-	100 ± 0 ^A^
125	0	0	0	0	0	8.33	8.33	0	16.66 ± 11.2 ^B^
62.5	0	0	0	0	0	8.33	0	0	8.33 ± 8.33 ^B^
31.2	0	0	0	0	0	0	0	0	0 ± ND ^B^
0	0	0	0	0	0	0	0	0	0 ± ND ^B^
LC_50_	154.32 (121.38–187.25) ppm								
***D. rerio* Embryos**									
**Treatment (ppm)**	**Time (h)**								
	**1 h**	**3 h**	**6 h**	**12 h**	**24 h**	**48 h**	**72 h**	**96 h**	**Total**
500	0	25	50	25	-	-	-	-	100 ± 0 ^A^
250	0	25	25	0	0	0	0	0	50 ± 12.9 ^B^
125	0	0	0	25	0	0	0	0	25 ± 11.2 ^BC^
62.5	0	25	0	0	0	0	0	0	25 ± 11.2 ^BC^
31.2	0	25	0	0	0	0	0	0	25 ± 11.2 ^BC^
0	0	0	0	0	0	0	0	0	0 ± ND ^C^
LC_50_	208.38 (152.48–264.28) ppm								

Results in adults represent the average of 12 measurements, while those in embryos are based on 16 measurements. Total results are shown ± standard error. Different letters indicate significant differences. LC_50_ is shown with its confidence intervals. ND stands for not determinable.

**Table 4:** Insecticidal activity of rosmarinic acid against *Spodoptera frugiperda* and *Tenebrio molitor*.

Treatment (ppm)	Larval Mortality (%)	
	*S. frugiperda*	*T. molitor*
1000	100 ± 0 ^A^	10 ± 6.88 ^A^
600	95 ± 5 ^A^	0 ± ND ^A^
400	85 ± 8.19 ^AB^	0 ± ND ^A^
160	55 ± 11.4 ^BC^	0 ± ND ^A^
80	45 ± 11.4 ^C^	0 ± ND ^A^
0	5 ± 5 ^D^	0 ± ND ^A^
LC_50_	176.81 (114.38–239.25) ppm	5256.28 (ND) ppm

Results are the average of 20 measurements ± standard error. Different letters indicate significant differences. LC_50_ is shown with its confidence intervals. ND stands for not determinable.

**Table 5:** Ecotoxicological effect of rosmarinic acid on *Poecilia reticulata* adults and fingerlings.

***P. reticulata* Adults**									
**Treatment (ppm)**	**Time (h)**								
	**1 h**	**3 h**	**6 h**	**12 h**	**24 h**	**48 h**	**72 h**	**96 h**	**Total**
500	0	0	0	0	0	0	0	0	0 ± ND
250	0	0	0	0	0	0	0	0	0 ± ND
125	0	0	0	0	0	0	0	0	0 ± ND
62.5	0	0	0	0	0	0	0	0	0 ± ND
31.2	0	0	0	0	0	0	0	0	0 ± ND
0	0	0	0	0	0	0	0	0	0 ± ND
LC_50_	ND								
***P. reticulata* Fingerlings**									
**Treatment (ppm)**	**Time (h)**								
	**1 h**	**3 h**	**6 h**	**12 h**	**24 h**	**48 h**	**72 h**	**96 h**	**Total**
500	0	0	0	0	16.66	0	8.33	0	25 ± 13.1 ^A^
250	0	0	0	0	0	0	8.33	8.33	16.66 ± 11.2 ^A^
125	0	0	0	0	0	0	8.33	0	8.33 ± 8.33 ^A^
62.5	0	0	0	0	0	0	0	0	0 ± ND ^A^
31.2	0	0	0	0	0	0	0	0	0 ± ND ^A^
0	0	0	0	0	0	0	0	0	0 ± ND ^A^
LC_50_	658.88 (338.75–979) ppm								

Results represent the average of 12 measurements. Total results are shown ± standard error. Different letters indicate significant differences. LC_50_ is shown with its confidence intervals. ND stands for not determinable.

**Table 6:** Ecotoxicological effect of rosmarinic acid on *Danio rerio* adults and embryos.

***D. rerio* Adults**									
**Treatment (ppm)**	**Time (h)**								
	**1 h**	**3 h**	**6 h**	**12 h**	**24 h**	**48 h**	**72 h**	**96 h**	**Total**
500	0	0	0	0	0	33.33	25	0	58.33 ± 14.9 ^A^
250	0	0	0	0	0	0	8.33	0	8.33 ± 8.33 ^B^
125	0	0	0	0	0	0	8.33	0	8.33 ± 8.33 ^B^
62.5	0	0	0	0	0	0	0	0	0 ± ND ^B^
31.2	0	0	0	0	0	0	0	0	0 ± ND ^B^
0	0	0	0	0	0	0	0	0	0 ± ND ^B^
LC_50_	463.81 (352.14–575.48) ppm								
***D. rerio* Embryos**									
**Treatment (ppm)**	**Time (h)**								
	**1 h**	**3 h**	**6 h**	**12 h**	**24 h**	**48 h**	**72 h**	**96 h**	**Total**
500	0	12.5	75	12.5	-	-	-	-	100 ± 0 ^A^
250	0	0	68.75	31.25	-	-	-	-	100 ± 0 ^A^
125	0	0	68.75	31.25	-	-	-	-	100 ± 0 ^A^
62.5	0	0	43.75	50	0	0	0	0	93.75 ± 6.25 ^A^
31.2	0	0	0		0	0	0	0	93.75 ± 6.25 ^A^
0	0	0	0		0	0	0	0	0 ± ND ^B^
LC_50_	21.42 (12.95–29.88 ppm)								

Results in adults represent the average of 12 measurements, while those in embryos are based on 16 measurements. Total results are shown ± standard error. Different letters indicate significant differences. LC_50_ is shown with its confidence intervals. ND stands for not determinable.

## Data Availability

The original contributions presented in this study are included in the article. Further inquiries can be directed to the corresponding author.
